# Monitoring the courtship flight trajectory of Latham's snipe (*Gallinago hardwickii*) using microphone arrays

**DOI:** 10.1002/ece3.9938

**Published:** 2023-03-31

**Authors:** Shiho Matsubayashi, Hideki Osaka, Reiji Suzuki, Kazuhiro Nakadai, Hiroshi G. Okuno

**Affiliations:** ^1^ Graduate School of Engineering Science Osaka University Toyonaka Japan; ^2^ toriR Lab Echizen Japan; ^3^ Graduate School of Informatics Nagoya University Nagoya Japan; ^4^ Department of Systems and Control Engineering, School of Engineering Tokyo Institute of Technology Tokyo Japan; ^5^ Graduate School of Informatics Kyoto University Kyoto Japan

**Keywords:** courtship flight, flight trajectory visualization, *Gallinago hardwickii*, localization, microphone arrays, robot audition

## Abstract

This study is the first to quantitatively measure the courtship display flights of Latham's snipe (*Gallinago hardwickii*), which is a “near threatened” species as of 2022 (IUCN red list of threatened species). By using a 16‐channel microphone array and 8‐channel microphone arrays, we localized the fine‐scale movements of courtship flights of one male performing at high altitude and high speed, and we estimated the direction from which each sound arrived using robot audition. Preliminary analyses of the azimuthal and elevation angles of the courtship flights partially revealed a fine‐scale flight trajectory. First, a male Latham's snipe gradually gained altitude while vocalizing sharp and harsh repeating calls, until it reached the flight peak altitude, then dove down while producing winnowing sound to the ground along the wetland zones without tall vegetation. This observation method is methodologically useful to establish a better understanding of Latham's snipe courtship flight site selection. Furthermore, this method can be extended to investigate other rare nocturnal or crepuscular birds that are too timid to risk ringing or tagging.

## Graphical abstract

This study is the first to quantitatively measure the courtship display flights of Latham's snipe (*Gallinago hardwickii*), using microphone arrays. We localized the fine‐scale movements of a male snipe performing courtship flights at high altitude and high speed. Preliminary analyses of the azimuthal and elevation angles partially revealed a fine‐scale trajectory of their courtship flight display.

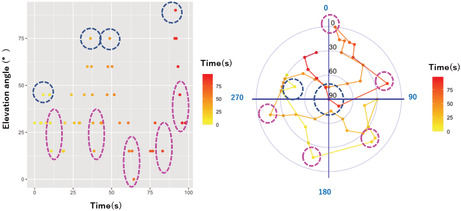

## 1 INTRODUCTION

Long‐distance migratory shorebirds are among the most threatened bird species across the globe, with 48% of known population exhibiting a declining trend. Latham's snipe (*Gallinago hardwickii*) is a species of shorebird that is found on the East Asian‐Australasian Flyway, the region with the highest proportion of shorebird populations. However, the information regarding population size and trends is limited (International Wader Study Group, 2003). These birds mainly breed on the grasslands or wetlands of Hokkaido Island—with occasional occurrences on the highlands of the main island of Japan (The Ornithological Society of Japan, 2012) and Far‐Eastern Russia, including southern Sakhalin (Hansen et al., 2020)—and then migrate to South‐Eastern Australia (The Ornithological Society of Japan, 2012). Their global population is estimated to be 30,000 (Hansen et al., 2016).

The international conservation status of Latham's snipe has been recently upgraded from “least concern” to “near‐threatened.” This is due to the extreme droughts in recent years in their wintering grounds in Australia where the population size is estimated to have declined by 70% (IUCN, 2022). In Australia, the species is being currently assessed under the threatened species list (Australian Government, 2022). On the breeding grounds in Japan, Latham's snipe is nearly threatened (The Ornithological Society of Japan, 2012) and listed as one among the 18 species that are at significant risk of future population decline. These observations are based on modeling data from three successive monitoring surveys conducted by the Japanese Ministry of the Environment for over 40 years (Amano et al., 2010). The rate of decline on the breeding site is most severe in the local population on the main island of Japan, which is located at a distance of over 600 km south of the core breeding population on Hokkaido island. For example, the number of individuals counted in 27 census sites in Tochigi prefecture decreased from 48 in 1986 to 14 in 2004, and this may be attributed to the loss of suitable habitat conditions (Hirano et al., 2005). Another census conducted in two main local breeding sites in the highlands of Nagano prefecture also revealed 16–21 males, which were observed in the 2008–2011 survey in Karuizawa area, and 21–23 males in 2011 in the Kirigamine area (Ishizuka & Hotta, 2012). Considering the uncounted individuals, this local population may face a high risk of extirpation that is measured according to the classic ecological threshold of 50, or an effective minimum population size that is necessary to prevent inbreeding depression (Franklin, 1980).

A regional decline was also observed at the core breeding site on Hokkaido island. For example, in a 2001 survey, Kitajima and Fujimaki (2003) observed that the average count decreased by 2.2 birds, compared to a survey in 1978–1991 wherein 38‐km^2^ transects were present in Tokachi plains. Furthermore, they investigated the effects of habitat alteration on the snipes' population trend and found that population decline was due to unfavorable conditions associated with agricultural fields (Kitajima & Fujimaki, 2003).

The accurate monitoring of the inhabitation status of birds and the assessment of their habitat use are important but challenging conservation tasks. Census is the most widely adopted approach for monitoring species composition, relative abundance, and habitat use in an area. Bird ringing has also been used to monitor the movements of birds. Recent technologies such as GPS loggers have been increasingly used to monitor the movements or biological parameters of birds as well as the environmental variables (Bouten et al., 2013). Although such tracking technologies have provided novel insights into the movements, behaviors, and ecology of birds, they have limited spatio‐temporal resolution and low‐recovery rates. Furthermore, they may not be ideal for the cases of relatively smaller birds mainly because of the potential effects of equipment size and weight (Bowlin et al., 2010). This is more apparent when the birds must perform delicate flight maneuverers at high speed, i.e., courtship display of Latham's snipe.

Recent advances in acoustic engineering, particularly, localization techniques based on microphone arrays, have resulted in increased popularity due to the massive potential for use in passive, low‐disturbance, and larger or longer spatiotemporal monitoring of sound‐producing animals including birds (Blumstein et al., 2011; Mennill, 2011). The localization technique quantifies the time delay associated with the arrival of the sound source that is detected via time‐synchronized microphones. This technique has been used to acoustically monitor microhabitat selection of the Boreal Chickadee (*Poecile hudsonicus*) and the Cape May Warbler (*Setophaga tigrine*), which are exhibiting a declining trend in boreal forests (Ethier & Wilson, 2020), occupancy of songbirds in the regeneration of reclaimed wellsite in boreal forests (Wilson & Bayne, 2019), territory of Mexican‐ant‐thrush (*Formicarius moniliger*; Kirschel et al., 2011), duetting behavior of rufous‐and‐white wrens (*Thryophilus rufalbus*; Mennill & Vehrencamp, 2008), and population density of ovenbirds (Dawson & Efford, 2009). However, despite their advantages, microphone arrays have not been widely adopted in the field mainly because of the limited availability of the required software and hardware.

To overcome these challenges, we previously developed HARKBird, a portable system for monitoring and analyzing birdsongs (Suzuki et al., 2017). The HARKBird consists of a standard laptop computer, an open‐source robot audition system called HARK (Honda Research Institute, Audition for Robots with Kyoto University; Suzuki et al., 2017), and commercially available low‐cost microphone arrays. According to the time delay in the arrival of sound sources derived from a microphone array, the HARKBird performs sound source localization and separation. The algorithm for sound source localization integrates multiple signal classification methods and spectrograms with a short‐time Fourier transformation (Nakadai et al., 2010). Localized sounds are separated into multiple songs in real‐time using the geometric high‐order decorrelation‐based sound separation method (Schmidt, 1986; see Nakadai et al., 2010 and Suzuki et al., 2017 for HARK and HARKBird, respectively). The software for HARKBird is entirely composed of a series of Python scripts with modules (e.g., wxPython and PySide) and other standard sound processing software (e.g., SOX, arecord, and aplay). All these software can operate in the Ubuntu 12.04 Linux operating system, and the latest HARK and HARK‐Python scripts can be installed from https://sites.google.com/view/alcore‐suzuki/home/harkbird.

We previously tested the spatial localization performance of the microphone arrays to monitor Great reed warblers (*Acrocephalus arundinaceus*) using 16‐channel microphone arrays in open riverbeds, where sound distortion because of vegetation is minimal. Our system estimated the location of two color‐banded individuals with a mean error distance of 5.5 ± 4.5 m from the location of observed song posts (Suzuki et al., 2017). We have acoustically monitored spatio‐temporal behaviors of songbirds such as Spotted Towhee (*Pipilo maculatus*; Sumitani et al., 2020), Japanese bush warbler (*Horornis diphone*; Suzuki, Matsubayashi, et al., 2018; Suzuki, Sumitani, et al., 2018), and zebra finches (*Taeniopygia castanotis*; Sumitani et al., 2021) through localization. Auditory observation using HARKBird has also proven to be effective in monitoring nocturnal and crepuscular species, such as courtship calls of Eurasian bittern (*Botaurus stellaris*; Matsubayashi et al., 2022), feeding behaviors and fledging of Ural owls (*Strix uralensis*), and territorial calls of the ruddy‐breasted crake (*Porzana Fusca*; Matsubayashi et al., 2021). In all the aforementioned cases, birds stayed at particular spots, i.e., song posts or nests, while vocalizing. The two primary objectives of this study are as follows. We first aim to investigate HARKBird's tracking capabilities of fine‐scale, high‐speed movements performed during courtship flights of Latham's snipes. We then showed that given replication, this method could provide fine‐scale information related to breeding habitat requirements.

## 2 MATERIALS AND METHODS

### 2.1 Study site

The study site was a subalpine, manmade, wet meadow in Nagano prefecture, Japan. Currently, only a few breeding grounds have been identified in Nagano. The documentation of the visits of Latham's snipes to the study site, which is approximately located at a distance of 60 km from the nearest known breeding ground, started only recently, and there is very little information regarding the size or breeding success of the population in this area. The wet meadow, which is currently abandoned, was originally constructed for a pasture connected by a nearby river, and other manmade wet meadows were created using irrigation pipes.

**FIGURE 1:**
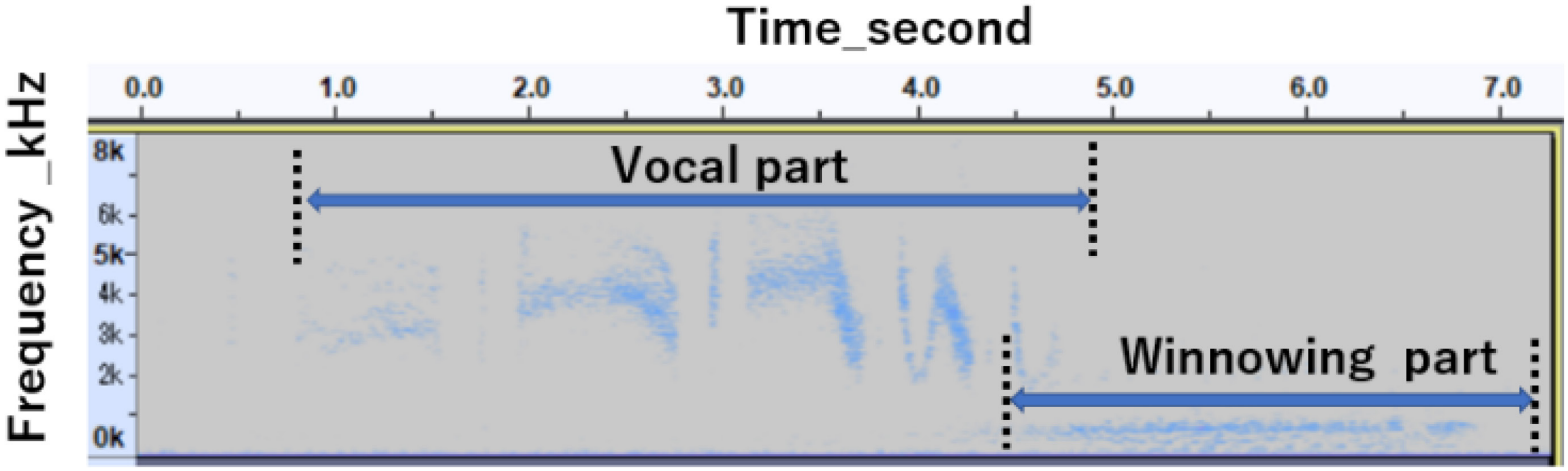
Spectrogram of vocal and non‐vocal parts of display flight of male Latham's snipe.

**FIGURE 2:**
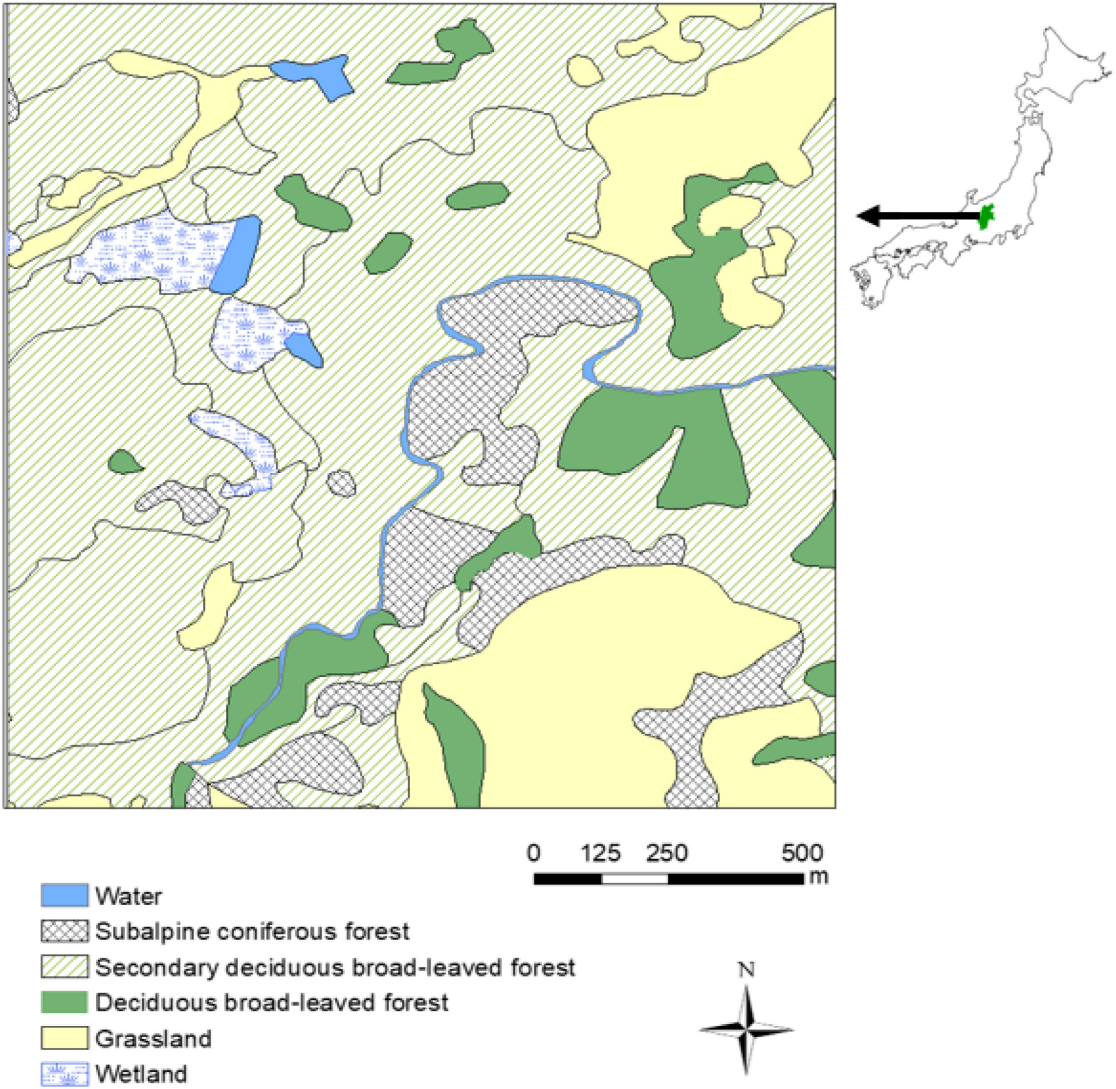
Habitat types of study area.

**FIGURE 3:**
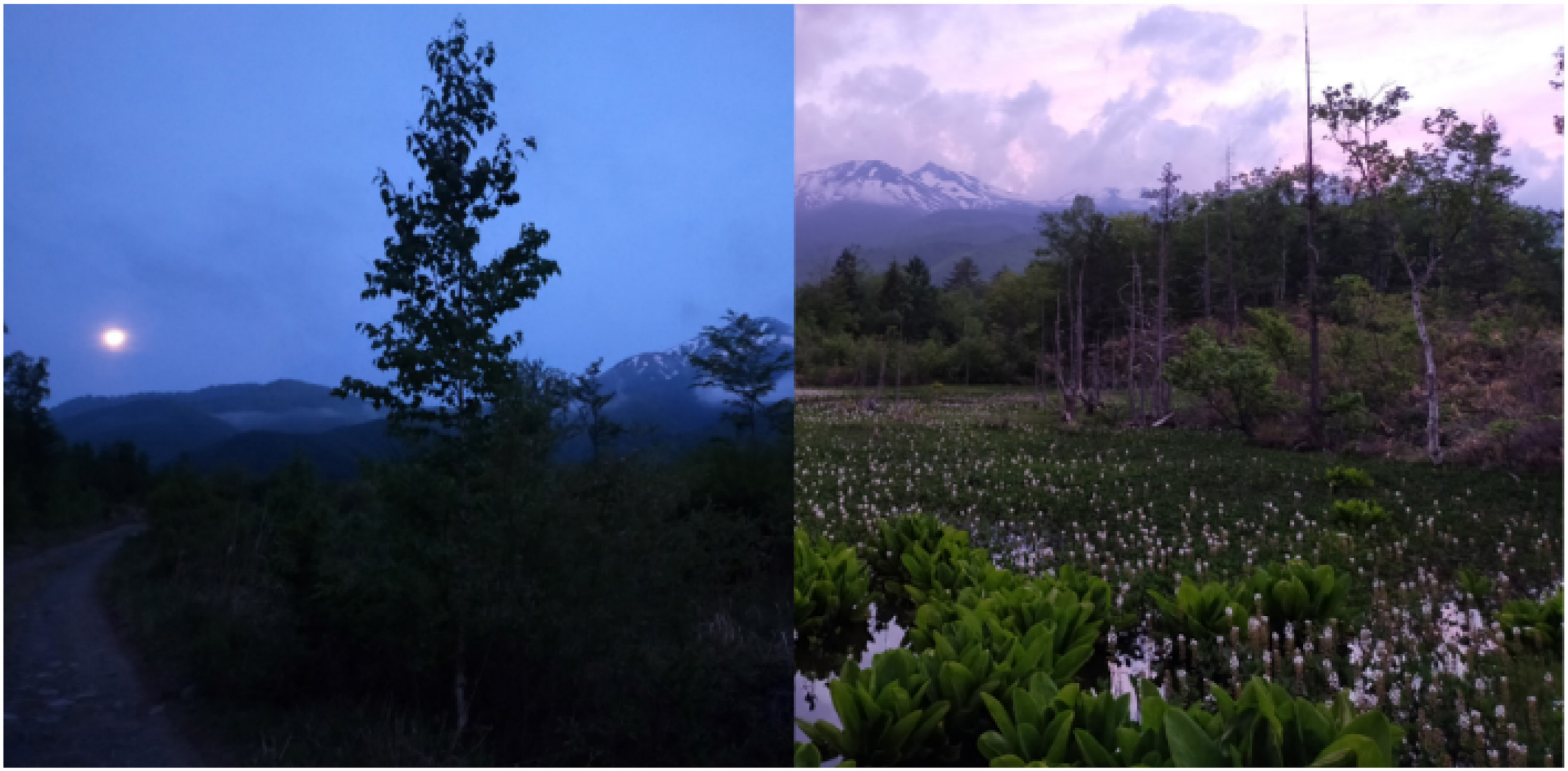
Photographs of study area at dawn (left) and dusk (right).

Figure 1 shows the land use of the study area, which was created using a map of scale 1/25,000 derived from the 6–7th vegetation survey conducted by the Biodiversity Center of Japan between 2000 and 2012 (Biodiversity Center of Japan, 2012). The site contains wetlands that are surrounded by subalpine conifer forests and mixed deciduous forests, mainly consisting of aspen (*Populus* spp.) and beeches (*Fagus* spp.) in various successional stages (Figure 2). The lowest part contains the stream and the wetlands. The water bodies of the wetland were covered with buckbean (*Menyanthes trifoliata*) and Asian skunk cabbage (*Lysichiton* spp.; Figure 3). A majority of the non‐forested parts of the study area were covered with grasses and shrubs of height 20–30 cm.

### 2.2 Human observers and bird recordings

The males of Latham's snipe perform courtship display flights while vocalizing sharp and harsh repeating calls (“zheep‐zheep‐zheep”) with an accelerating frequency when gaining altitude, which later becomes “zubie‐zubie‐zubie” at an approximate peak elevation of the flight. They then produce a winnowing thrust (“za‐za‐za‐za‐za”) that is generated by the outer tail feathers while diving down to the ground (Video S1). Figure 2 shows an actual spectrogram of the vocal and non‐vocal sounds that are visualized using Audacity version 3.0.0. Although their display calls are most active before dawn and after dusk (Nakamura & Shigemori, 1990), we visually and auditorily confirmed the approximate location of display flights a few hours before or after dawn and dusk to avoid the Asian black bear (*Selenarctos thisbetanus*) in the study area.

**FIGURE 4:**
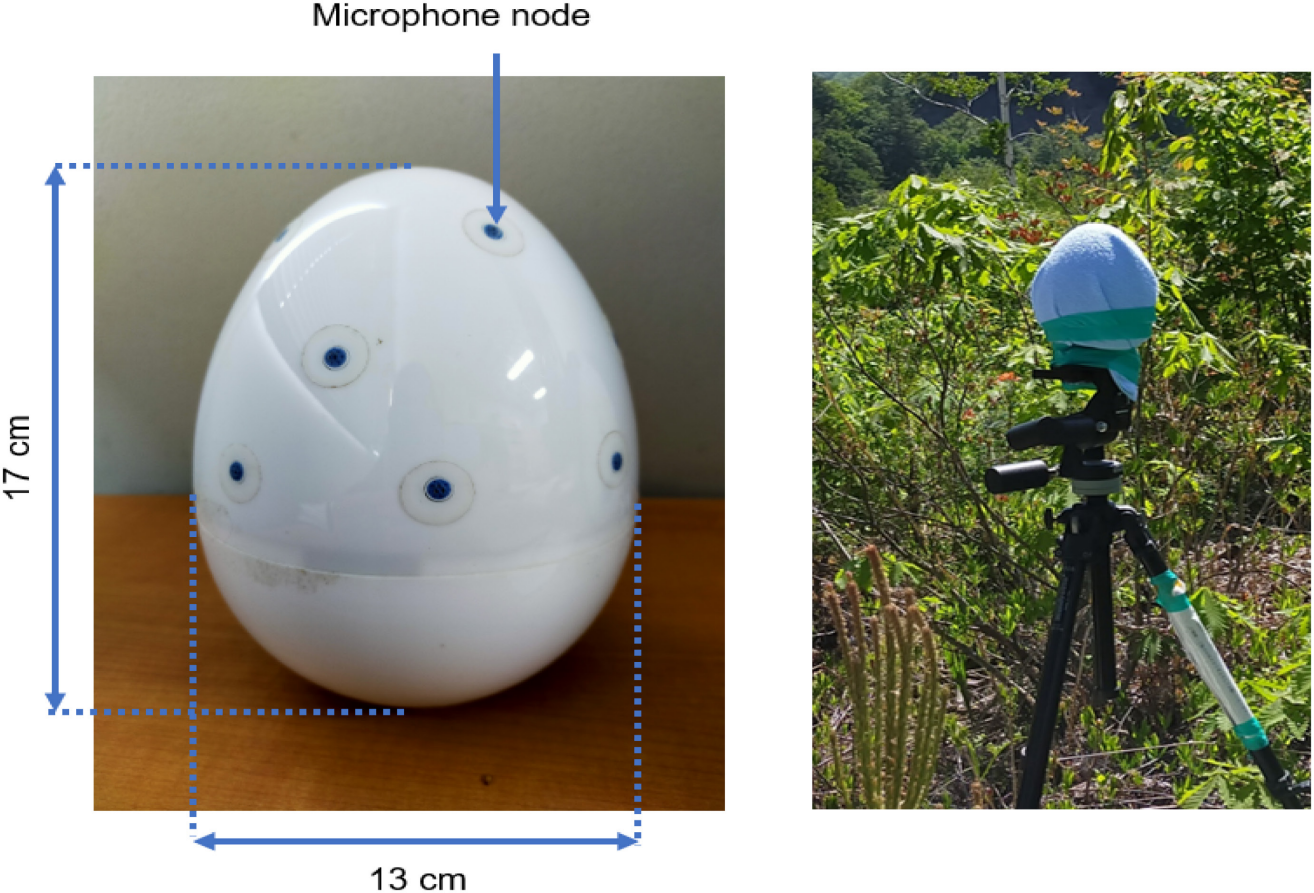
Geometry of 16‐channel microphone array from side view (left) and top view of the microphone on a tripod in the field (right). Black dots on the body of the microphone array indicate placement of microphone node.

We recorded Latham's snipes from 17:00 on June 6th to 10:00 on June 7th, 2020 using a 16‐channel circular microphone array and two 8‐channel microphone arrays. The 16‐channel microphone array was used as a stand‐alone and water‐resistant type (height 17 cm; width 13 cm) comprising 16 microphone nodes arranged in three tiers on an egg‐shaped frame (Figure 4, DACHO, System in Frontier). About 8 of the 16 microphone nodes were arranged horizontally at every 45° angle along the periphery of the egg‐shaped body. The other eight nodes were arranged horizontally in two tiers above the bottom tier, with four nodes in each tier at every 90° angle around the periphery. This three‐tiered structure helped estimate the direction of arrival (DOA), both in the elevational DOAs and the azimuthal DOAs. On the other hand, the 8‐channel microphone array (height 12 cm; width 8 cm) consisted of eight microphone nodes that were arranged in one tier on an egg‐shaped frame at every 45° angle around the periphery, which helped estimate the azimuth DOAs (TAMAGO‐03, System in Frontier). We placed each microphone array (with a cover for protection from the wind) on a tripod at a distance of approximately 1.5 m above the ground, and we connected it to a small PC (Raspberry‐pi 4) and a mobile battery using USB cables. We synchronized all the PCs by tethering them to a cell phone. We then silently monitored the birds in a non‐invasive manner, away from potential nesting sites, without playback experiments using loudspeakers to trigger territorial responses.

### 2.3 Localization using HARKBird and its performance

**FIGURE 5:**
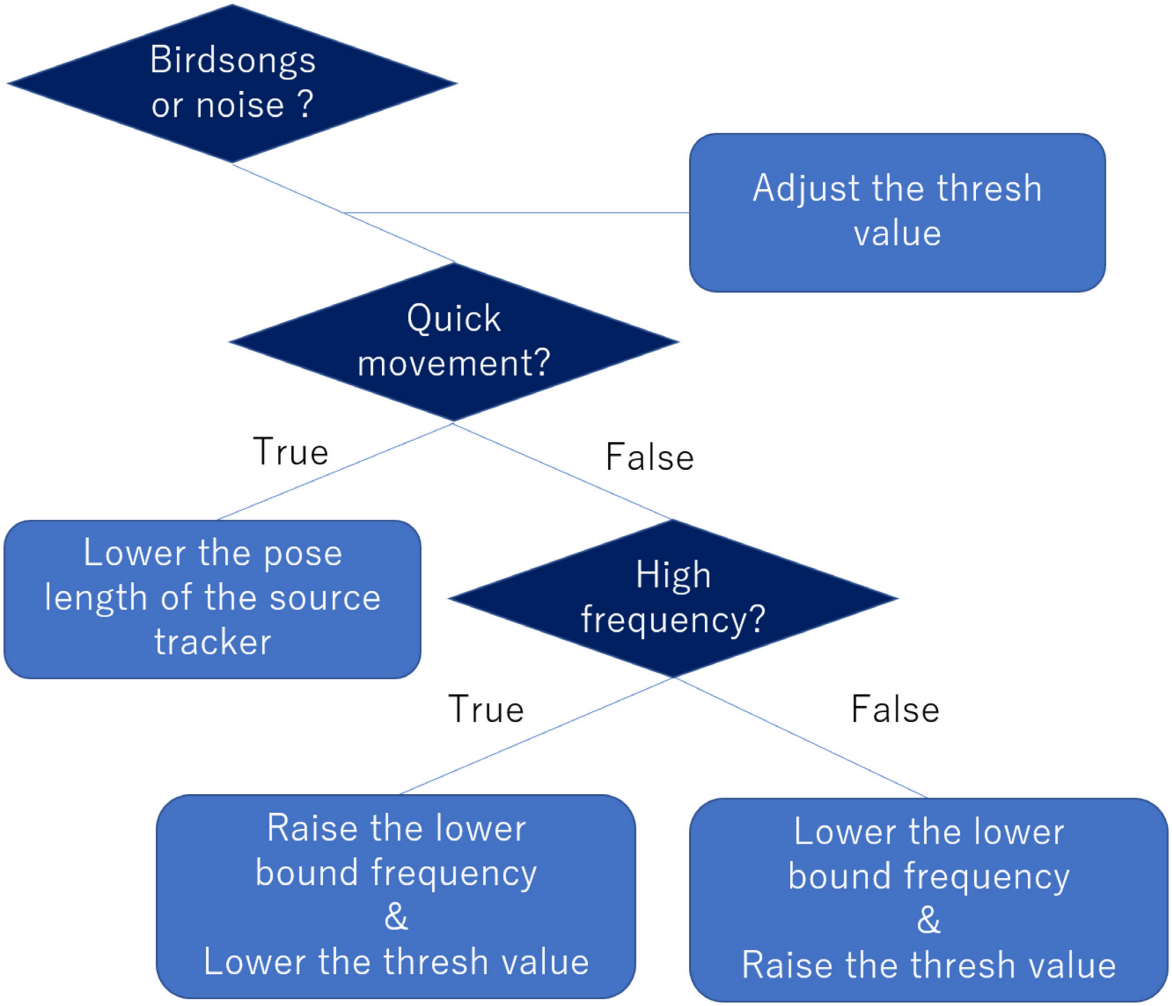
Flowchart showing process of adjusting the parameters in HARKBird.

We estimated the DOA of the sound source, which was acquired offline from the microphone array and analyzed with HARKBird using a flowchart (Figure 5). Because of the size of the recordings, we localized sounds every 15 min. The major localization parameters of HARKBird are summarized in Table 1. We inspected all localized sounds auditorily and manually removed localized sounds that were not generated by snipes. Other sounds in the soundscape stemmed from biological sources, e.g., common (*Cuculus canorus*) to non‐biological sound, e.g., streams and winds.

**TABLE 1:** Localization parameters.

Parameter	Unit	Value	Explanation
PAUSE_LENGTH	ms	300	Previous section length of sound source
MIN_SRC_INTERVAL	Degree	10	Threshold for angle difference to be regarded as the same sound source
PERIOD	Number of frames	10	Cycle to calculate the localization results
THRESH		28	Threshold to separate the sound source and noise
UPPER BOUND FREQUENCY	Hz	5000	Upper bound frequency
LOWER BOUND FREQUENCY	Hz	800	Lower bound frequency
NUM_SOURCE		1	Number of sound sources

We examined temporal localization performance according to the following procedure. We first manually annotated the timing of vocal and non‐vocal parts of display flights by visually inspecting spectrograms and auditory inspection of sounds. We then calculated the total duration of sound. We also calculated temporal localization performance by taking the ratio of localized duration derived by HARKBird and total duration of sound annotated. We estimated the two dimensional (2D) spatial position of sound sources by triangulating DOAs that were derived from a pair of microphone arrays thereafter.

## 3 RESULTS

### 3.1 Direct observation

**FIGURE 6:**
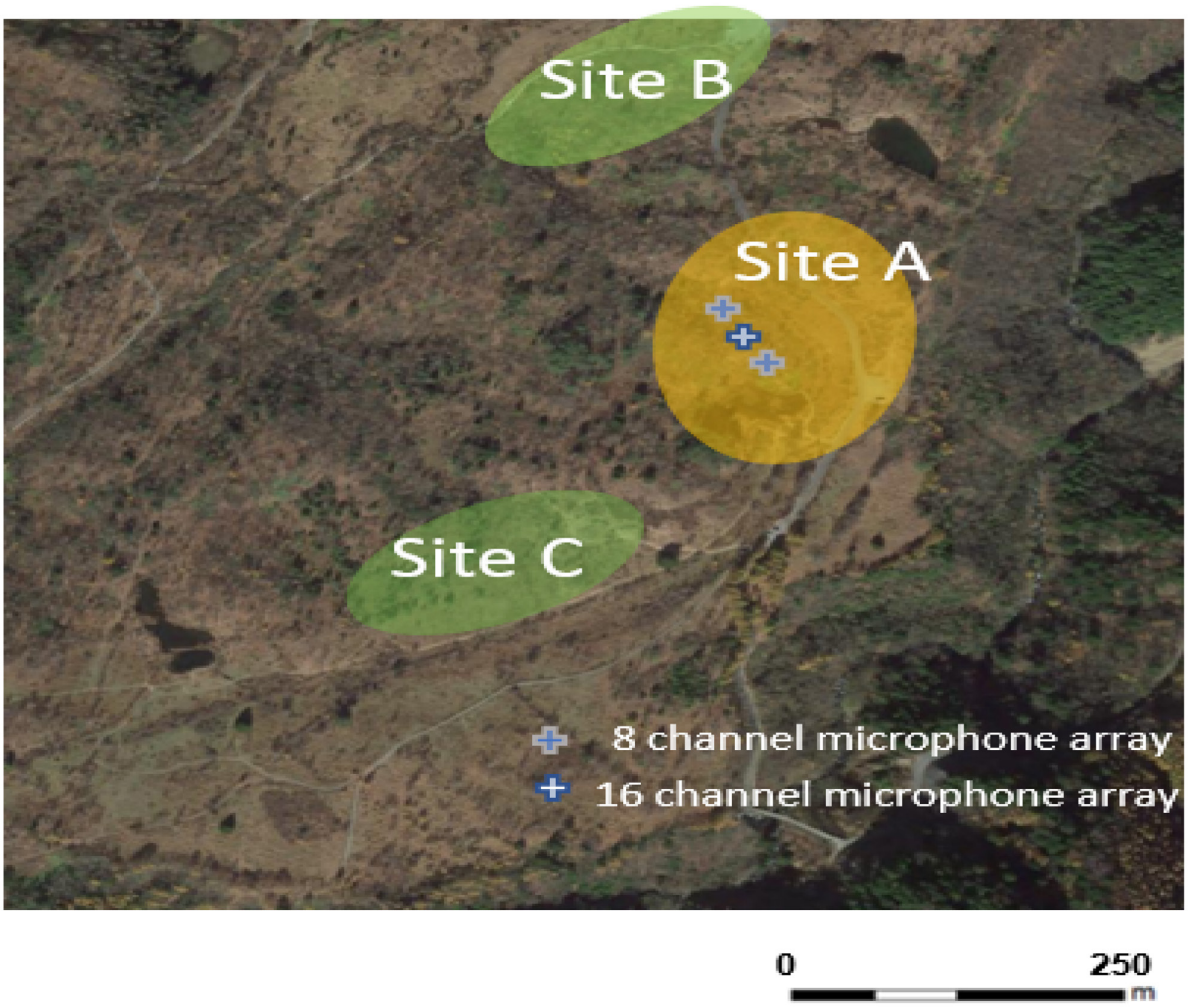
Locations of confirmed display sites A, B, and C and microphone arrays.

We visually and auditorily confirmed three display sites; the location of each site and location of each microphone array are shown in Figure 6. At site A, an individual of unidentified sex began calling “*bee‐bee‐bee*” at 18:30 (approximately 30 min before sunset) on a song post within a shrub adjacent to a wetland area, which could not be seen by observers. Shortly before sunset, male snipes began their display flights. They gradually flew higher in a quick, zigzag manner until they reached an altitude of approximately 50–70 m above the ground. They then rapidly dove toward the ground. At the end of the ‘almost vertical’ dive at approximately 5–10 m above the ground, the birds suddenly turned horizontally and began making quick zigzag movements over the tree canopies, and they then flew high up in the air.

During this routine—when the birds repeated the zigzag movements and dove down—they appeared to circulate and trace similar flight trajectories. The flight was always unidirectional, from east to west, at site A during direct human observation. At site A, another individual approached from the southwest direction relative to the observer and seemed to join or invade the course of the display flights. However, we could not confirm whether it was a female attempting to copulate with the male at site A or another male that flew in from site C.

### 3.2 Number of localized display flights

**FIGURE 7:**
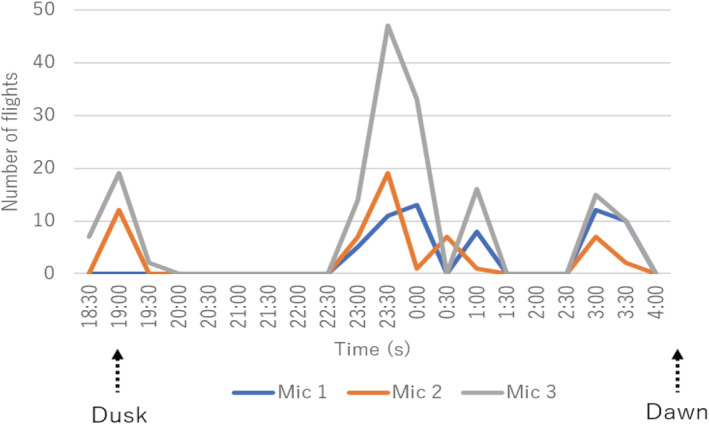
Number of display flights of male during the midnight peak at site A. Three lines indicate number of flights localized by three 8‐channel microphone arrays at site A.

We detected 163 flights of a single male in 18:30–4:30 through one of the microphones placed at the northern edge of site A where we placed microphone arrays. As shown in Figure 7, the number of display flights showed a cyclic pattern with four peaks: 9:00 (dusk), 22:30–0:00 (midnight), 0:30–1:30, and 2:30–4:00 (dawn).

### 3.3 Localization performance

We examined the temporal localization performance for 95 flights during the midnight peak in site A between 23:15 and 00:30, which was derived from a microphone array that recorded the largest number of flights. The average temporal localization performance was 85.26%. The relative position of the bird and the microphone array significantly affected the localization performance. When the bird was in close proximity, according to the intensity of sounds, 95% of 40 display flights were localized between 10:30 and 11:30. When the bird receded from the microphone array between 11:30 and 00:30, the localization performance decreased to 78.2% of the 55 display flights.

### 3.4 Time series variation of descending flight trajectories

**FIGURE 8:**
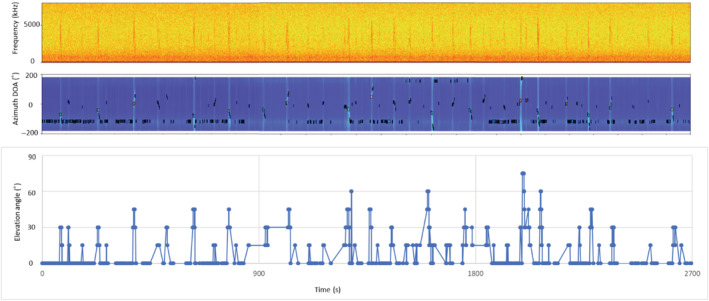
Spectrograms (top) and localized elevation angles (bottom) of display flights of male derived from 16‐channel microphone array in 45 min during midnight peak at site A.

Figure 8 shows the azimuth and elevation angles during the midnight peak lasting for 45 min at site A from 23:15 to 00:30. The greater values of the elevation angles and sound intensity indicate that the bird flew closest to the microphone array at 0:10:40 at an elevation angle of 60°. During this time frame, the prelude part of the display flight, or sharp and harsh repeating calls (“*zheep‐zheep‐zheep*”) that the bird vocalized when gaining altitude in a zigzag manner, was not recorded. The absence of this part indicates that the bird attained sufficient altitude, and this was beyond the distance limit of our microphone arrays and thus could not be localized when the bird reached the descending point above or near the microphone array. Changes in azimuth angles in display flights also showed that the bird consistently started diving down in the north of the microphone array in either the eastward or westward direction.

### 3.5 Azimuthal and elevation angle localization of courtship flights

**FIGURE 9:**
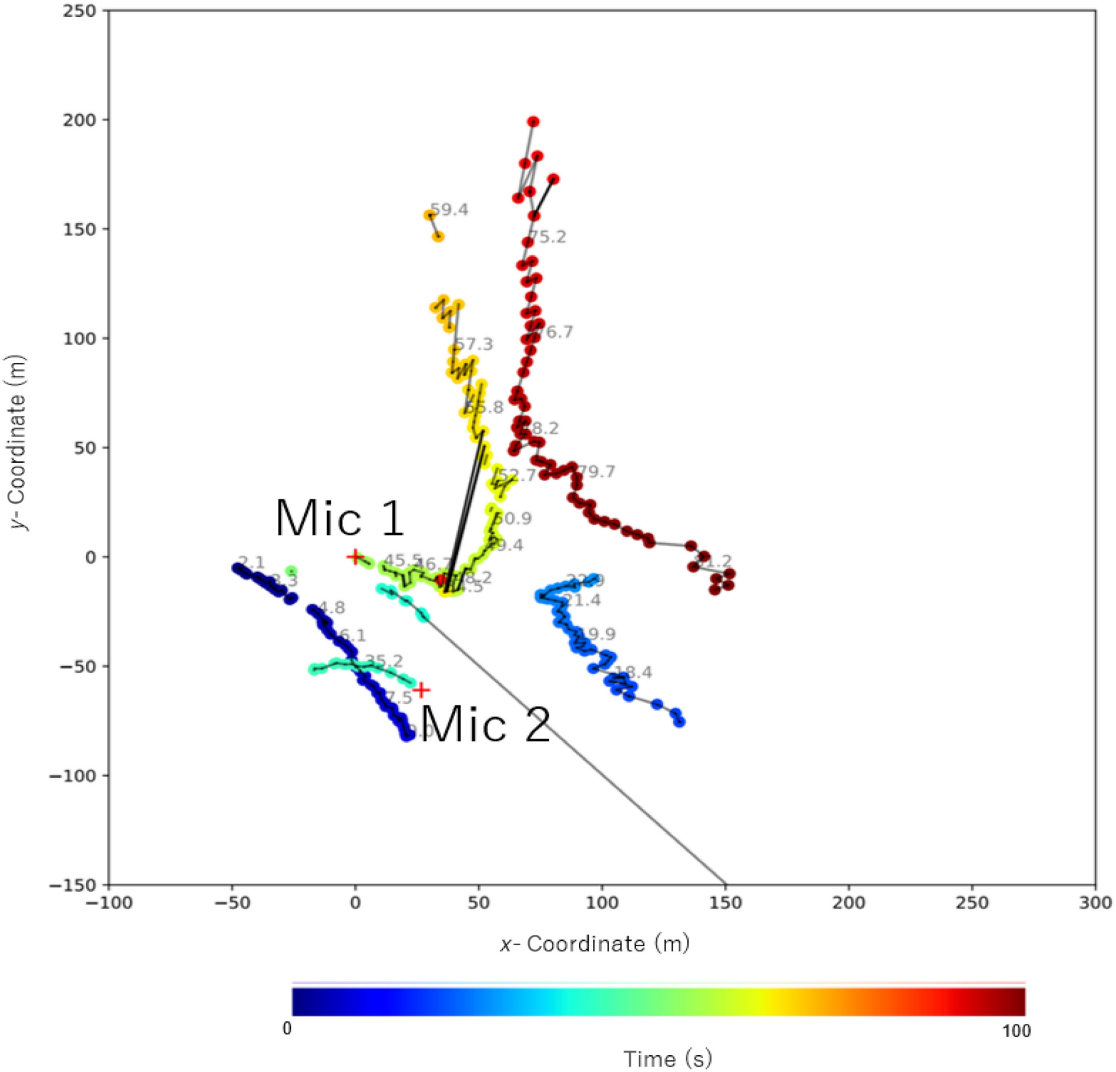
Sample flight trajectory of male in 2D based on azimuth DOAs derived from two 8‐channel microphone arrays at site A.

The 2D flight trajectories based on azimuthal DOAs derived from two 8‐channel microphone arrays that were separated by a distance of 66.7 m, at 19:26 are shown in Figure 9. The bird started the first display of flight 25 m west of Mic 1, vocalizing the prelude part consisting of a series of sharp “*zheep‐zheep‐zheep*” sound followed by “*zubie‐zubie‐zubie*….” sound, from north to south. Then, it dove down while winnowing. The snipe started the second display flight immediately after the 1st flight on the same trajectory line from north to south, which is shown in blue, and it flew beyond the distance limit of localization. The next instance at which the bird was localized was when it crossed the midpoint of the two microphone arrays from west to east, while calling the prelude part again. The location of the snipe was estimated only when this bird was localized by two microphone arrays. The results of the 2D localization show that the distance limit of localization of our microphone arrays was in the range of 250–300 m.

**FIGURE 10:**
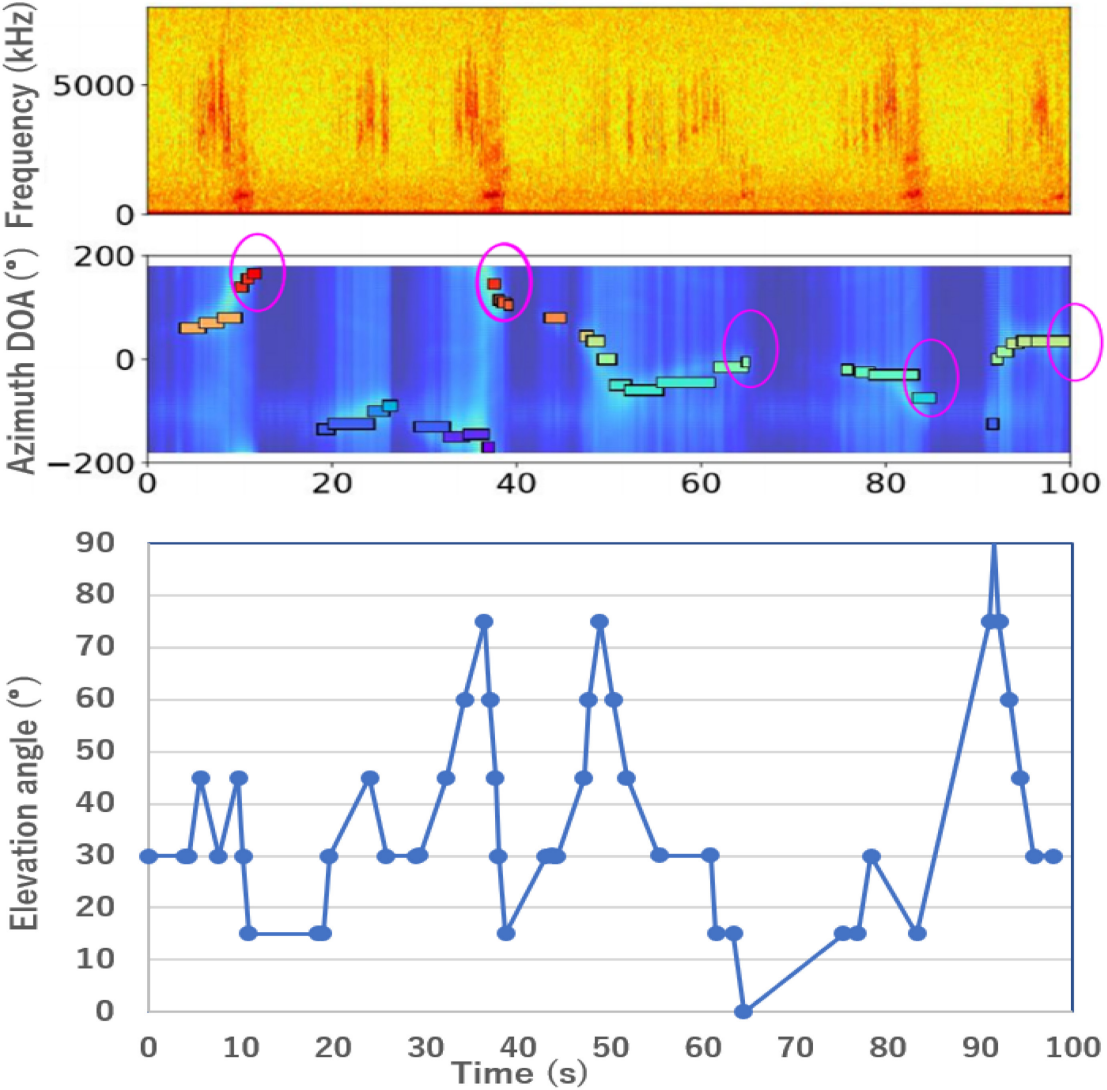
Spectrogram of display flight (top), azimuthal angle (middle), and elevation angle (bottom) of display flight as a function of time. Sudden drops in flight elevation are indicated by pink circles.

The example flight trajectory measured using azimuth, and the elevation angles of a male display flight derived from a 16‐channel microphone array are shown in Figure 10. In this example, starting at 19:26, the bird performed courtship flight five times in 100 s. In each display flight, the bird gained altitude by a rapid zigzagging flight while vocalizing the prelude part that sounds similar to “*zie‐ zie‐zie*…” for several seconds until it reached the altitudinal peak of the display flight. This prelude was vocalized relatively fast, and it consisted of two sound elements in 1 s. Once the bird reached the peak altitude of the flight, it swept down while producing two different calls. It first vocalized heavy and rhythmically harsh calls that sounded like “*zubie‐zubie‐zubie*….” This part was relatively slow and heavy, and it consisted of a single sound element, “*zubie,”* which lasted for 2 s. On the way down to the ground, the bird started to winnow while generating sound consisting of sound elements, “*za‐za‐za‐za‐za‐za‐za‐za.”*

These flights occurred in all directions except for the northwest of the microphone array, which was blocked by tree canopies. The lowest point at the deepest part of the dive was recorded at 63 s from the beginning of the recording, when the bird dove down in the southwest direction of the microphone array, right above the wetland. This corresponded to a 0° elevation angle at a distance of 1.5 m above the ground. After this dive, the bird was silent for 10 s before it began the next call. It was observed that the bird gained altitude when it vocalized again, preparing for the subsequent winnowing calls (Figure 6).

**FIGURE 11:**
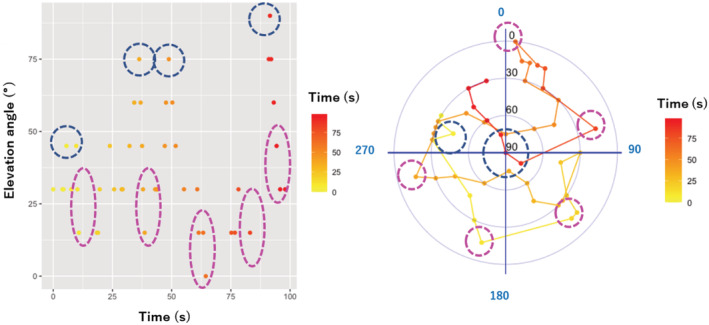
Localized elevation angles (left), and flight trajectory of display flight (right). Flight trajectory was constructed based on elevation angle (black on the vertical axis) and azimuthal angle (blue on the outer edge of the circle) derived from the microphone array. Elevations peaks and bottoms of display flight are shown in blue and pink dashed circles, respectively. DOA, direction of arrival.

A part of the flight trajectory during the same time frame is shown in Figure 11. It should be noted that this flight trajectory was constructed based on the azimuthal and elevation angles that were derived from a single microphone array. Thus, the position of each point on the figure represents the bird's position relative to the microphone array at the center of the circle. The inward and outward lines indicate that the bird was flying toward and away from the microphone array, respectively. Based on the amplitude of the sound, we believe that the bird was closest to the microphone array at the first winnowing part of the display flight.

In this recording, the bird reached the highest peak of the display flight while vocalizing a series of heavy “*zubie*” sounds, which is a part of its heavy and rhythmically harsh vocalization, four times, as indicated by the blue dashed circles in Figure 7. The first peak was localized at an elevation angle of 45°, which was lower than the three subsequent peaks. Among these, two peaks were localized at an angle of 75° and the last one was localized at an elevation angle of 85° almost directly above the microphone array. Upon reaching the peak of each flight, the bird dove down while changing the direction of flight several times instead of going into a straight descent.

## 4 DISCUSSION

### 4.1 Merits of microphone arrays for rare bird surveys

This study showed how microphone arrays can be used to extract detailed information regarding the courtship displays of moving individual birds, with minimum disturbance to the target animal. The four main advantages of using microphone arrays for the conservation of rare and sound‐producing birds are as follows. First, it provides an accurate distance from the location of the bird. Estimating the distance becomes difficult when the object is far away and moving fast, and the difficulty is further increased when visibility is limited, i.e., in a dense forest, or under low light conditions. Under these conditions, detection is primarily based on auditory cues. However, the results of distance estimation via auditory survey are not free from bias associated with human observers. For example, field experiments on the distance estimation skills of six experienced observers based on auditory detection suggested that observers could not differentiate distances beyond 65 m (Alldredge et al., 2007). The results of localization in our field experiment revealed that the distance limit in the azimuth direction of 8‐channel microphone arrays was approximately 250 m, revealing flight trajectories of a snipe based on DOAs that were derived from two microphone arrays. The localized results also outperformed human observers in tracking the elevation angles of these fast‐moving birds.

Second, it collects data in an unobtrusive manner based only on sounds. The remote surveying of auditory behaviors without using external devices such as GPS loggers significantly reduces the potential costs – both the logistical costs of observing target birds and the potential stress caused to them. Attaching external devices results in a significant increase in the energy expenditure independent of birds' attributes such as sex, age, and body mass, which leads to a decline in survival rates or changes in behaviors (Barron et al., 2010). Passive monitoring also allows the collection of birds' behavioral data that are not affected by nearby human observers.

Third, it provides fine‐scale analyses of the spatio‐temporal behaviors of the target birds. Our previous efforts targeted monitoring bird vocalizations, i.e., courtship, feeding, or fledging behaviors of nocturnal Ural owls (*Strix uralensis*) and territorial calls of the ruddy‐breasted crake (*Porzana Fusca*; Matsubayashi et al., 2021) were performed with little movement. This study revealed that the monitoring system can effectively track the movements of targets in terms of the aforementioned behaviors at a much higher speed. Owing to the birds' wide range of movements at high speed, which exceeds the range within which the microphone arrays can collect sounds, the results of the 2D localization may be a partial representation of the birds' flight trajectory. Although limited, the localization results—according to both azimuthal and elevation angles—revealed fine scale movements in courtship flights that could not be visually detected. These results revealed how the Latham's snipe selects preferred courtship sites within potential sites, in association with habitat properties.

The fourth advantage is the reproducibility of these data. The time‐stamped data with location information were useful in reexamining the spatio‐temporal flight patterns of birds. The results have indicated that the flight had spatial periodicity measured in elevation angles, tracing similar trajectories each time. These data were also useful for investigating the location and timing of particular types of sound – that is, calls or winnows – generated during display flights.

### 4.2 Potential impacts of vegetation change on courtship of Latham's snipe

The study area is not a known breeding site for Latham's snipe. The reason why this species has recently started using this site has been a subject of discussion. Although it is unclear whether their visits in recent years are occasional, we suspect that the habitat heterogeneity that is created by vegetative structure and composition, comprising abandoned pasture surrounded by shrubs and secondary forests, provides suitable courtship grounds for the snipes. The significance of habitat heterogeneity for the snipe's breeding ground is also discussed by Kitajima and Fujimaki (2003), wherein they stated that a decline in local population was observed in agricultural fields or croplands and pasture in Hokkaido. We also hypothesize that the current vegetative structure also provides favorable conditions to perform display flights. The display flights of Latham's snipes are known to peak on full moon days (Iida, 1995). The night we observed the bird was a quiet full moon period, which is a preferred courtship conditions for the bird. The analyses of the flight trajectory showed that when diving down while winnowing, the Latham's snipe males selectively swept down to a lower altitude over the wetland area without thick and tall vegetation, possibly to maximize their chances of sending their courtship cues. The absence of vegetative obstacles may further result in an increase in the efficiency of females in receiving both visual and auditory signals.

However, the abandoned pasture within the study area faces rapid plant succession by the reduced water supply from irrigation pipes. The absence of grazing pressure from cattle further advances plant succession, thus converting the grassland to shrubs and secondary forests. The thick and tall vegetation of secondary forests may reduce the visibility as well as the transmittance of the winnowing flights, thus limiting the females' ability to assess the quality of the males.

### 4.3 Limitations and future directions

Three technical limitations were identified in this study. First, an optimization support function for HARKBird is required. Localization success depends on many factors, including the characteristics of birdsongs—for example, the amplitudes, frequencies, complexities, bird behaviors, various ambient noises associated with the surrounding environment, and the distance between the microphone array and the recording target. Currently, we locate birds by manually adjusting the localization parameters separately for each situation using a trial‐and‐error process using a general framework (Figure 8). This tuning process is intuitive and black‐boxed, and thus, users can neither finely tune each parameter nor directly compare the results after adjusting each parameter. Adopting an interactive interface where users can optimize the localization parameters (Sugiyama et al., 2015) will result in a significant rise in the efficiency of localizing target birds.

Second, we estimated elevation angles of courtship flights derived from a single microphone array. By using multiple microphone arrays, we can estimate the flight height on the *x*–*y* coordinates. This time‐stamped location data derived from multiple microphone arrays help calculate the flight speed. Furthermore, it is also possible to draw a complete flight trajectory of each male, identify territorial boundaries, and track how each male changes its auditory behavior or flight trajectory in the presence of potential mates or male intruders to his territory, if a wider tract of land using more microphone arrays was surveyed.

Third, the automatic sound classification scheme should be refined. In this study, localized sounds were inspected manually. By using an automatic classification scheme, we can minimize the cost of manual inspection. This will also increase the efficiency of data analysis in investigating when each sound type (vocal or non‐vocal winnowing) occurred during the display flight.

## 5 CONCLUSION

This study shows how microphone arrays can collect detailed spatial information on behaviors of fast‐moving rare birds with minimum disturbance to the target. We auditorily monitored display flights of the near‐threatened species, Latham's snipe, on their breeding ground at night. With replication, the flight trajectories, in association with the surrounding vegetation and other habitat types, could reveal important microhabitat requirements for this near‐threatened species. Based on the preliminary analyses, we suspect that males use the 2D and three‐dimensional (3D) space in a wetland area by adjusting the course of the flight path; that is, the birds choose an open area to perform winnowing dives, while using more cluttered vegetation for nesting sites. To conserve such rare species, multiple microphone arrays may be adopted in future studies to gain a better understanding of fine‐scale habitat conservation and restoration for this species with specific and difficult‐to‐observe breeding habitat requirement.

## AUTHOR CONTRIBUTIONS

**Shiho Matsubayashi:** Conceptualization (equal); data curation (equal); formal analysis (lead); funding acquisition (equal); investigation (lead); methodology (equal); validation (lead); visualization (equal); writing – original draft (lead); writing – review and editing (lead). **Hideki Osaka:** Conceptualization (equal); data curation (supporting); formal analysis (supporting); investigation (supporting); resources (equal); writing – review and editing (supporting). **Reiji Suzuki:** Conceptualization (equal); funding acquisition (equal); methodology (equal); resources (lead); software (lead); visualization (equal); writing – original draft (equal); writing – review and editing (equal). **Kazuhiro Nakadai:** Conceptualization (equal); funding acquisition (equal); resources (equal); software (lead). **Hiroshi G. Okuno:** Resources (lead); software (lead); writing – original draft (supporting); writing – review and editing (supporting).

## CONFLICT OF INTEREST STATEMENT

All authors declare that the research was conducted in the absence of any commercial or financial relationships that could be construed as a potential conflict of interest.

## Supporting information


Video S1

## Data Availability

When accepted, the sound data presented in this paper will be available upon request.
